# Analytical theory and possible detection of the *ac* quantum spin Hall effect

**DOI:** 10.1038/s41598-017-05452-4

**Published:** 2017-07-11

**Authors:** W. Y. Deng, Y. J. Ren, Z. X. Lin, R. Shen, L. Sheng, D. N. Sheng, D. Y. Xing

**Affiliations:** 1 National Laboratory of Solid State Microstructures and Department of Physics, Nanjing University, Nanjing, 210093 China; 2 Department of Physics, South China University of Technology, Guangzhou, 510640 China; 3 Collaborative Innovation Center of Advanced Microstructures, Nanjing University, Nanjing, 210093 China; 4 Department of Physics and Astronomy, California State University, Northridge, California, 91330 USA

## Abstract

We develop an analytical theory of the low-frequency *ac* quantum spin Hall (QSH) effect based upon the scattering matrix formalism. It is shown that the *ac* QSH effect can be interpreted as a bulk quantum pumping effect. When the electron spin is conserved, the integer-quantized *ac* spin Hall conductivity can be linked to the winding numbers of the reflection matrices in the electrodes, which also equal to the bulk spin Chern numbers of the QSH material. Furthermore, a possible experimental scheme by using ferromagnetic metals as electrodes is proposed to detect the topological *ac* spin current by electrical means.

## Introduction

Topological insulators (TIs) are currently on the research front of condensed matter physics, because of their fundamental interest and potential applications in spintronic devices^1–13^. Two-dimensional (2D) TIs are also called the quantum spin Hall (QSH) systems, as they can host the interesting QSH effect, in which quantized spin current or spin accumulation can be generated in response to an applied electric field. A QSH system is an insulator in the bulk with a pair of conducting gapless edge states traversing the bulk band gap^1–3^. The *Z*_2_ invariant^14^ or spin Chern numbers^15–17^ have been proposed to describe the QSH systems. The *Z*_2_ invariant is well-defined only in the presence of time-reversal (TR) symmetry^14^, which is consistent with the fact that the edge states are gapless when the TR symmetry is present, and usually gapped otherwise. While the spin Chern numbers are found to be equivalent to *Z*_2_ invariant for TR-invariant systems, their robustness does not rely on any symmetries^16–18^. The nonzero spin Chern numbers guarantee that the edge states must appear on the sample boundary, which could be either gapped or gapless, depending on symmetries or local microscopic structures of the sample edges^19^. The edge states will become Anderson localized in the presence of TR symmetry breaking and disorder. As a consequence, the QSH effect is often unstable in realistic environments. Up to now, conductance through edge channels near the theoretically predicted quantized value has been detected in small samples of HgTe quantum wells^20^ and InAs/GaSb bilayers^21^.

Recently, *ac* spin-dependent electronic transport has started to capture attention, stimulating the emerging field of *ac* spintronics. Jiao and Bauer theoretically predicted that the *ac* voltage signal is much larger than the *dc* one, and could be used to detect spin currents in the spin pumping transport^22^. Wei *et al*. found experimentally that the *ac* spin current is much larger than the *dc* component in a ferromagnet-normal junction with time-dependent magnetization vector^23^. In a recent work^24^, the QSH effect driven by an *ac* electric field was studied numerically, by using the Kubo linear-response formula. It was demonstrated that the *ac* QSH effect exhibits quite different properties from the intensively researched *dc* QSH effect^24^. In particular, the *ac* QSH effect was found to be stable to random magnetic disorder, which breaks both spin conservation and TR-symmetry^24^.

In this paper, we show that the basic characteristics of the low-frequency *ac* QSH effect can be interpreted in terms of single-parameter adiabatic spin pumping. The time dependence in the driving electric field is essential for generating *ac* spin current flowing from the bulk of the QSH sample to an electrode. By using the well-established time-dependent scattering matrix formalism, the *ac* spin Hall conductivity is linked to the winding numbers of the reflection matrices in the electrode, which also equal to the spin Chern numbers of the QSH material. Our theory indicates that while the *ac* and *dc* QSH effects behave quite differently, they share the same topological origin. We further show that when ferromagnetic metals are used as electrodes, the topological *ac* spin current will induce an electrical voltage difference along the electrodes, suggesting a possible experimental way to observe the *ac* QSH effect by electrical means.

## Results

### A General Description

**Figure 1:**
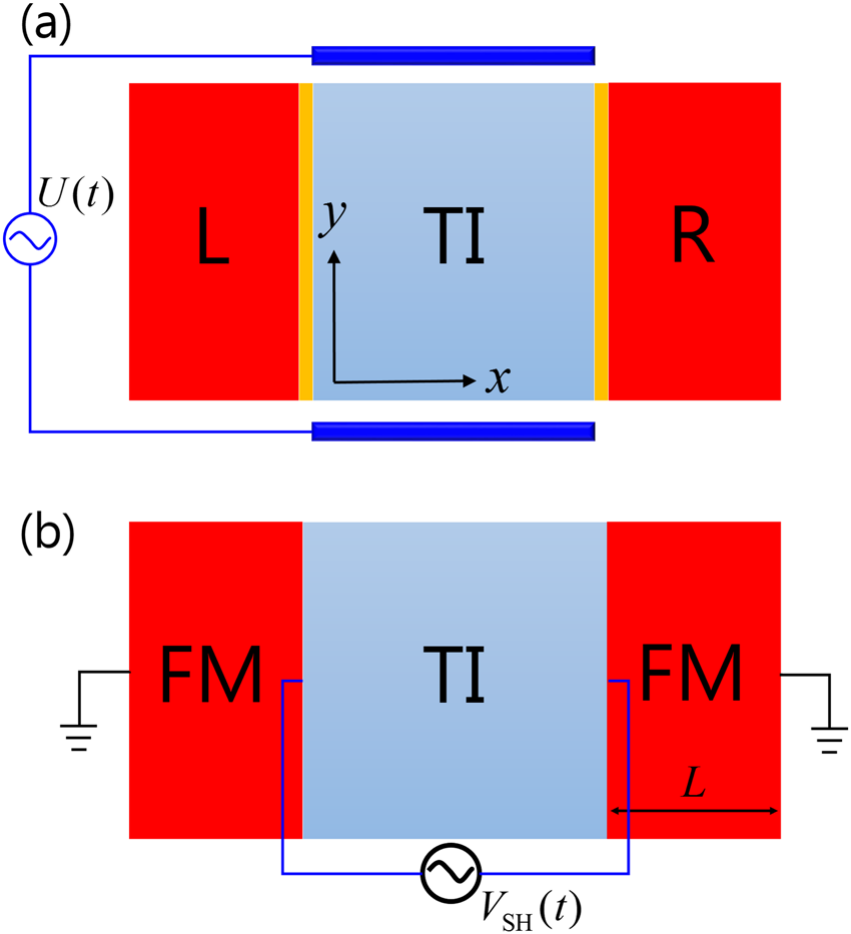
A schematic view of a proposed setup to study the topological *ac* QSH effect. (**a**) A 2D QSH material is placed between two conductive plates (blue). When an *ac* electrical voltage *U*(*t*) is applied across the plates, an electric field *E*(*t*) will be generated in the *y* direction. In response, an *ac* Hall spin current *j*_*s*_(*t*) is created along the *x* direction. The QSH material is attached with the left and right metallic electrodes (red), with potential barriers (yellow) in between. (**b**) When ferromagnetic metals with length *L* are used as the electrodes, an *ac* electric voltage difference *V*_SH_(*t*) can be induced between the inside edges of the two electrodes, suggesting a possible experimental way to detect the *ac* QSH effect electrically.

Let us consider the setup illustrated in Fig. 1(a). A QSH sample is placed between two conductive plates. When an *ac* electric voltage difference *U*(*t*) is applied to the plates, an *ac* electric field *E*(*t*) = *E*_0_ cos(*ωt*) is established along the *y* direction between the two plates. The induced Hall spin current *j*_*s*_(*t*) will flow along the *x* direction. The QSH sample is attached with the left and right electrodes, which serve as source and drain electrodes for the Hall spin current. In this proposed setup, if open boundary conditions are used in the *y* direction, edge states will appear near the upper and lower edges of the sample. The effective Hamiltonian of the edge states is ${H}_{{\rm{edge}}}={v}_{{\rm{F}}}{k}_{x}{s}_{z}{\tau }_{z}$, where the Pauli matrices *s*_*z*_ and *τ*_*z*_ represent electron spin and the two sample edges, respectively. Since the Hamiltonian *H*_edge_ does not depend on *k*_*y*_, and the vector potential of the electric field has only nonzero *y* component, the vector potential does not couple to the edge states, meaning that the edge states do not contribute to the spin pumping. Therefore, we can demonstrate the significant difference between the *ac* and *dc* QSH effect. To facilitate our general discussion, we assume that the *ac* electric field also exists in the electrode and barrier. This assumption does not change the topological properties of the system, and will not affect the main conclusion. The size of the system is taken to be sufficiently large, so that we can neglect any finite-size effects and employ a periodic boundary condition in the *y* direction. As a result, the Hamiltonian of the system of the QSH sample and electrodes takes the form $H({k}_{x},{\tilde{k}}_{y},x)$ with ${\tilde{k}}_{y}={k}_{y}-eA(t)$. Here, (*k*_*x*_, *k*_*y*_) is the 2D momentum, where ${k}_{x}=\frac{\hslash }{i}\frac{\partial }{\partial x}$ is an operator, and *k*_*y*_ is a good quantum number. $A(t)=-\frac{{E}_{0}}{\omega }\,\sin (\omega t)$ is the vector potential of the *ac* electric field $E(t)={E}_{0}\,\cos (\omega t)$. In the TI region, $H({k}_{x},{\tilde{k}}_{y},x)$ is the Hamiltonian of the QSH material with a band gap, while in the electrodes, it represents the Hamiltonian of a metal.

The electron Fermi energy is set to be in the band gap of the QSH material. We consider first the ideal case, where the electron spin is conserved. We consider the right electrode, and the situation in the left electrode is similar. Let ${\hat{r}}_{ss}({\tilde{k}}_{y})$ be the reflection matrix for an electron with spin *s* (↑ or ↓) at the Fermi level transmitting from the electrode toward the QSH material. In the adiabatic regime, the spin current density pumped into the electrode at time *t* can be evaluated by using the scattering matrix formula^25, 26^

1 $${j}_{s}(t)=\frac{1}{{L}_{y}}(\frac{\hslash }{4\pi i})\sum _{{k}_{y}\in {\rm{BZ}}}\,{\rm{Tr}}({\hat{r}}_{\uparrow \uparrow }^{\dagger }\frac{d}{dt}{\hat{r}}_{\uparrow \uparrow }-{\hat{r}}_{\downarrow \downarrow }^{\dagger }\frac{d}{dt}{\hat{r}}_{\downarrow \downarrow }),$$

with *L*_*y*_ as the width of the QSH sample in the *y* direction. We focus on the adiabatic pumping regime, where the frequency *ω* of the *ac* electric field is much smaller than the bulk energy gap Δ_gap_ of the QSH material^27^. We notice that the reflection matrices depend on *t* only through the variable ${\tilde{k}}_{y}={k}_{y}-eA(t)$, such that $\frac{d}{dt}{\hat{r}}_{\uparrow \uparrow }=eE(t)\frac{d}{d{k}_{y}}{\hat{r}}_{\uparrow \uparrow }$, and $\frac{d}{dt}{\hat{r}}_{\downarrow \downarrow }=eE(t)\frac{d}{d{k}_{y}}{\hat{r}}_{\downarrow \downarrow }$. By using these relations and replacing the summation over *k*_*y*_ in Eq. (1) by an integral, we derive the *ac* spin Hall conductivity, defined as ${\sigma }_{{\rm{SH}}}(\omega )={j}_{s}(t)/E(t)$, to be

2 $${\sigma }_{{\rm{SH}}}(\omega )=\frac{e}{4\pi }({W}_{\uparrow }-{W}_{\downarrow }),$$

where

3 $${W}_{s}=\frac{1}{2\pi i}{\int }_{{\rm{BZ}}}\,d{k}_{y}\,{\rm{Tr}}\,({\hat{r}}_{ss}^{\dagger }\frac{d}{d{k}_{y}}{\hat{r}}_{ss}).$$

In the left electrode, the *ac* spin Hall conductivity has an opposite sign to Eq. (2).

Since the electron Fermi energy is in the band gap of the QSH material, an electron incident from the electrode will be fully reflected, and the reflection matrices must be unitary, i.e., ${\hat{r}}_{ss}^{\dagger }({\tilde{k}}_{y}){\hat{r}}_{ss}({\tilde{k}}_{y})=\hat{1}$. Besides, they are periodic functions of *k*_*y*_ in a Brillouin zone. As a result, one can identify immediately *W*_*s*_ as winding numbers, which are always integers (see Appendix 1). Therefore, while the spin Hall conductivity *σ*_SH_(*ω*) is defined as the ratio between two time-dependent quantities, i.e., the *ac* spin current *j*_*s*_(*t*) and *ac* electric field *E*(*t*), it is integer-quantized at any time, in units of the spin conductivity quantum $\frac{e}{4\pi }$, in the adiabatic regime. Very often, continuous models are employed in theoretical works. In a well-defined continuous model, the electron wave functions should be continuous in the ${k}_{y}\to \pm \infty$ limit, in order to maintain a similar periodic boundary condition as in the Brillouin zone in a lattice model. This implies

4 $$\mathop{\mathrm{lim}}\limits_{{k}_{y}\to +\infty }\,{\hat{r}}_{ss}({\tilde{k}}_{y})=\mathop{\mathrm{lim}}\limits_{{k}_{y}\to -\infty }\,{\hat{r}}_{ss}({\tilde{k}}_{y}\mathrm{)}.$$

Under this condition, *W*_*s*_ remain to be integer-quantized. In the example considered later, we will see that *W*_*s*_ in fact equal to the spin Chern numbers of the QSH system. When small spin-mixing perturbations, such as the Rashba spin-orbit coupling, are present, spin-flip reflection processes will occur with small probabilities, and the *ac* spin Hall conductivity will deviate from the integer-quantized value in a gradual manner, similarly to the *dc* QSH effect.

We need to point out that the *ac* Hall spin current originates from the time dependence of the Hamiltonian caused by the *ac* applied electric field, as clearly indicated by Eq. (1). One might think that by taking the limit *ω*→0, the conclusion for the *ac* QSH effect should be applicable to the *dc* QSH effect, which is not true. For an exactly static electric field (*ω* = 0), since one can choose an electrostatic scalar potential to make the Hamiltonian independent of time, no spin current can be generated in the setup shown in Fig. 1(a). At this time, the spin current *j*_*s*_(*t*) = 0 since the derivative of the reflection matrix with respect to time is zero in Eq. (1). Therefore, the *ac* QSH effect is substantially different from the *dc* QSH effect. Moreover, the above general discussion about the *ac* QSH effect does not rely on any symmetries, which is also different from the *dc* QSH effect. As we know, the bulk of the TI is an insulator, where the electron transport involves the evanescent modes in the band gap, as shown in Appendix 2, similarly to what happens in the famous Thouless charge pump. The *ac* QSH effect is linked to the bulk topological invariant of the QSH system, as seen in the concrete example below, which is an intrinsic property of the bulk electron wave functions. As a result, the *ac* QSH effect is a bulk transport phenomenon, being robust against TR-symmetry breaking and disorder^24^.

### A Concrete Example

As a concrete example, we consider the Bernevig-Hughes-Zhang (BHZ) model, which can be used to describe the HgTe quantum wells^28^ or InAs/GaSb bilayers^29^. The BHZ model Hamiltonian reads

5 $${H}_{{\rm{QSH}}}={v}_{{\rm{F}}}({k}_{x}{\hat{s}}_{z}{\hat{\sigma }}_{x}-{\tilde{k}}_{y}{\hat{\sigma }}_{y})-({M}_{0}-B{\tilde{k}}^{2}){\hat{\sigma }}_{z}.$$

Here, we retain the $B{\tilde{k}}^{2}$ term with ${\tilde{k}}^{2}={k}_{x}^{2}+{\tilde{k}}_{y}^{2}$, as it ensures that the condition Eq. (4) is fulfilled, and the topological properties of the system are properly defined. In fact, the spin Chern numbers of this model, given by ${C}_{\uparrow (\downarrow )}=\pm \frac{1}{2}[{\rm{sgn}}({M}_{0})+{\rm{sgn}}(B)]$ ^30^, are dependent of *B*. Other nonessential nonlinear terms of momentum in the original model have been neglected for simplicity.

The *ac* QSH effect, as a topological transport phenomenon, is insensitive to the material details of the electrodes. We model the electrodes by using a simple parabolic Hamiltonian ${H}_{{\rm{E}}}=-{U}_{0}+\frac{{\tilde{k}}^{2}}{2m}$. *U*_0_ is taken to be large compared with all other energy scales, so as to guarantee that the electrodes have sufficient number of conducting channels for the spin current to flow through. Finite potential barriers of height *V*_0_ and thickness *d* exist at the interfaces between the QSH material and electrodes, similar to the setup considered in ref. 31. By following the same procedure detailed in ref. 31, linearizing the Hamiltonians of both the QSH material and electrodes with respect to *k*_*x*_, one can obtain for the reflection coefficients (see Appendix 2)

6 $${r}_{\uparrow \uparrow }({\tilde{k}}_{y})=-\frac{\cos (\theta )+i[{\rm{sh}}({\gamma }_{0}d)-\,\sin (\theta )\,{\rm{ch}}({\gamma }_{0}d)]}{{\rm{ch}}({\gamma }_{0}d)-\,\sin (\theta )\,{\rm{sh}}({\gamma }_{0}d)},$$

and ${r}_{\downarrow \downarrow }({\tilde{k}}_{y})={{r}_{\uparrow \uparrow }({\tilde{k}}_{y})|}_{\theta \to (\pi -\theta )}$, where ${\gamma }_{0}=\frac{{V}_{0}}{\hslash }\sqrt{2m/{U}_{0}}$ and $\theta ={\rm{\arg }}[{v}_{{\rm{F}}}{\tilde{k}}_{y}+i({M}_{0}-B{\tilde{k}}_{y}^{2})]$.

**Figure 2:**
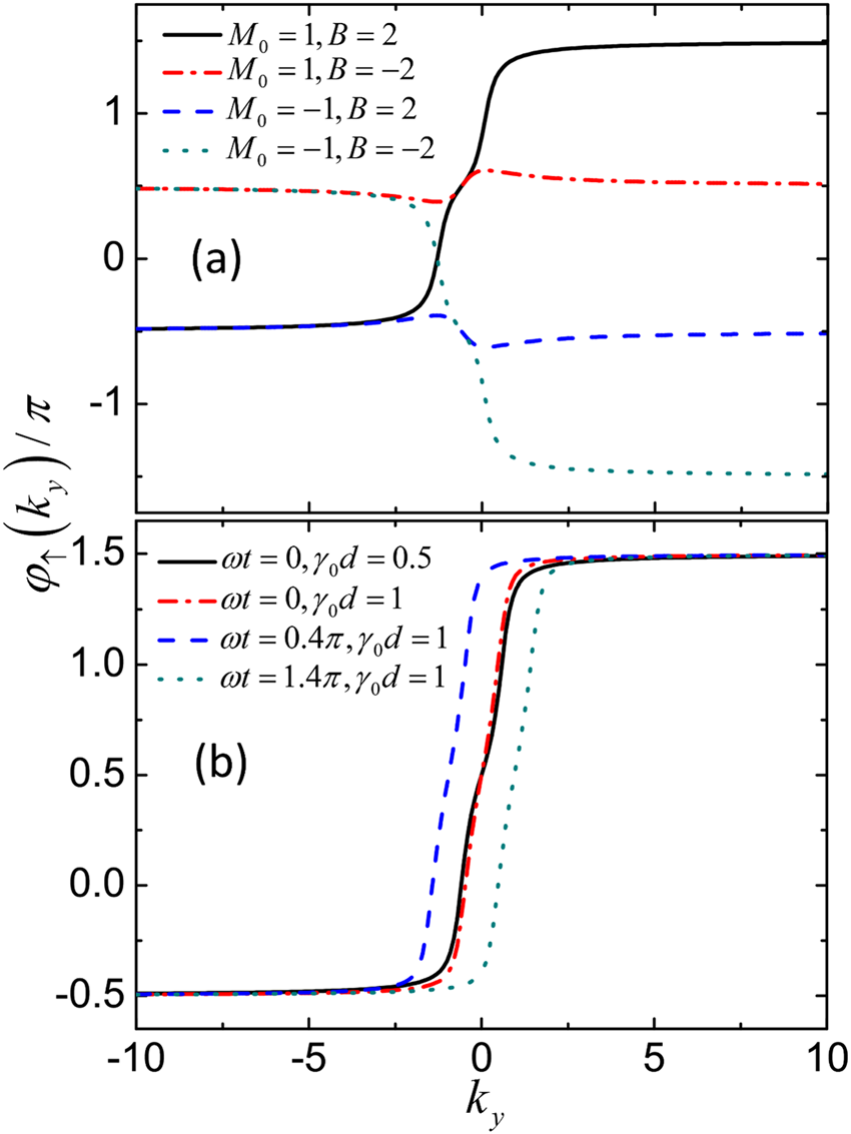
Argument of the complex reflection amplitude, ${\phi }_{\uparrow }({k}_{y})={\rm{\arg }}\,({r}_{\uparrow \uparrow })$, as a function of *k*_*y*_, for (**a**) four different sets of (*M*_0_, *B*), and (**b**) four sets of (*ωt*, *γ*_0_ *d*). The other parameters are taken to be *eE*_0_/*ω* = 1, and (**a**) *ωt* = *γ*_0_ *d* = 0, (**b**) *M*_0_ = 1, *B* = 2. The unit set is *v*_F_ = |*M*_0_| = 1.

For the present model, the reflection matrix ${\hat{r}}_{ss}({\tilde{k}}_{y})$ is simply a number, satisfying ${|{r}_{ss}|}^{2}=1$. Therefore, with changing *k*_*y*_ from −∞ to ∞, ${r}_{ss}({\tilde{k}}_{y})$ keeps traveling on the unit circle around the origin on the complex plane, and forms a closed orbit due to single-value condition Eq. (4). The quantity *W*_*s*_ defined in Eq. (3) is the winding number of the closed orbit around the origin. For convenience, the winding number can also be expressed as

7 $${W}_{s}=\frac{1}{2\pi }[{\phi }_{s}(\infty )-{\phi }_{s}(-\infty )],$$

where *φ*_*s*_(*k*_*y*_) is the argument of ${r}_{ss}({\tilde{k}}_{y})$. In the absence of the potential barrier, i.e., *γ*_0_ *d* = 0, the reflection amplitude Eq. (6) reduces to ${r}_{\uparrow \uparrow }({\tilde{k}}_{y})=-{e}^{-i\theta }$. The winding number can be determined by tracking how the argument *φ*_*s*_(*k*_*y*_) of ${r}_{ss}({\tilde{k}}_{y})$ evolves with changing *k*_*y*_ from −∞ to ∞. In Fig. 2(a), we plot four different representative behaviors of *φ*_↑_(*k*_*y*_). For simplicity, we have chosen the unit set, where *v*_F_ = |*M*_0_| = 1. From Fig. 2(a), we see that if *M*_0_ > 0 and *B* > 0, *φ*_↑_(*k*_*y*_) increments 2*π* with varying *k*_*y*_ from −∞ to ∞. If *M*_0_ < 0 and *B* < 0, *φ*_↑_(*k*_*y*_) decrements 2*π*. In the other cases, where *M*_0_ > 0 and *B* < 0, or *M*_0_ < 0 and *B* > 0, *φ*_↑_(*k*_*y*_) does not change. The behaviors of *φ*_↓_(*k*_*y*_) can be analyzed similarly. Consequently, we obtain from Eq. (7) the following expression for the winding numbers

8 $${W}_{\uparrow (\downarrow )}=\pm \frac{1}{2}[{\rm{sgn}}({M}_{0})+{\rm{sgn}}(B)]\equiv {C}_{\uparrow (\downarrow )}.$$

Interestingly, the winding numbers are exactly equal to the spin Chern numbers of the BHZ model. As expected, the *ac* spin Hall conductivity is integer-quantized, ${\sigma }_{{\rm{SH}}}=[{\rm{sgn}}({M}_{0})+{\rm{sgn}}(B)]\frac{e}{4\pi }\equiv ({C}_{\uparrow }-{C}_{\downarrow })\frac{e}{4\pi }$, which is consistent with the numerical result calculated from the Kubo theory at low frequencies^24^. This relation indicates that while the *ac* and *dc* QSH effects behave quite differently, they share the same topological origin.

For a nonvanishing potential barrier, i.e., *γ*_0_ *d* > 0, the condition |*r*_*ss*_|^2^ = 1 is still satisfied. This means that with changing *k*_*y*_ from −∞ to ∞, the reflection amplitudes ${r}_{ss}({\tilde{k}}_{y})$ always move on the unit circle around the origin on the complex plane. As a result, the winding numbers cannot change values with changing *γ*_0_ *d*. In other words, Eq. (8) remains valid for a nonvanishing potential barrier. Here, based upon the same topological argument, we may also get some insight into why the *ac* spin Hall conductivity ${\sigma }_{{\rm{SH}}}(\omega )={j}_{s}(t)/E(t)$ is integer-quantized, being independent of time. At a given time *t*, the system Hamiltonian *H*(*t*) has some small deformation from *H*(*t* = 0). As long as the difference *H*(*t*) − *H*(0) is not large enough to close the band gap, the winding numbers and spin Hall conductivity are unchangeable. In Fig. 2(b), we plot the argument *φ*_↑_(*k*_*y*_) of ${r}_{\uparrow \uparrow }({\tilde{k}}_{y})$ for some different sets of *γ*_0_ *d* and *ωt* in the case *M*_0_ > 0 and *B* > 0. We see that while its curve deforms with changing *γ*_0_ *d* or *ωt*, *φ*_↑_(*k*_*y*_) always increments 2*π*, independent of the barrier strength or time. For essentially the same reason, it is easy to understand that whether the *ac* electric field exists in the electrode does not affect the expressions for the winding numbers and *ac* spin Hall conductivity. The winding numbers may change values, only if the bulk band gap in the QSH material closes. In this case, the condition |*r*_*ss*_|^2^ = 1 of full reflection no longer holds, and the trajectories of ${r}_{ss}({\tilde{k}}_{y})$ can sweep across the origin on the complex plane, which leads to a change in the winding numbers, signaling a topological phase transition.

**Figure 3:**
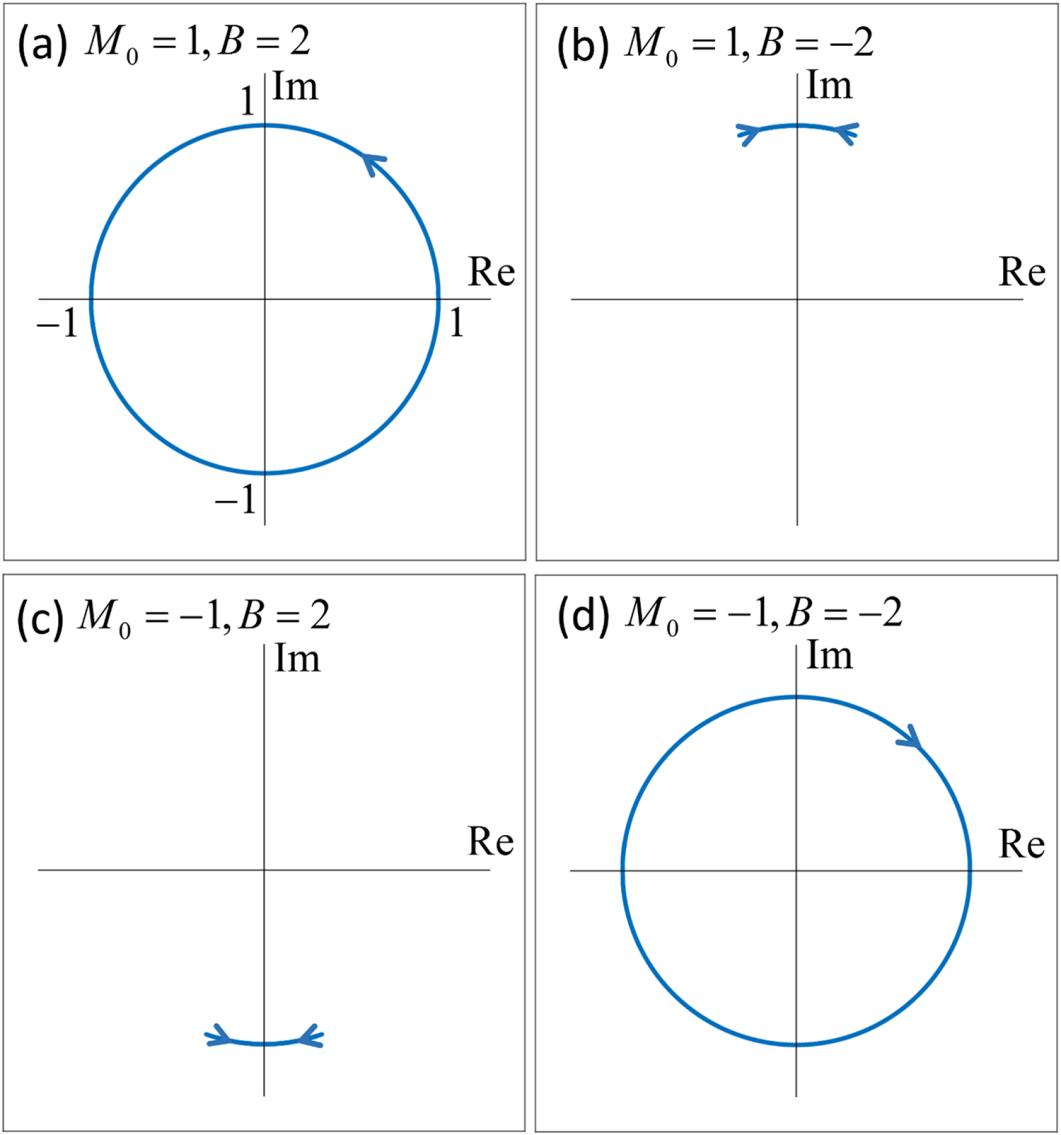
Trajectories of the reflection amplitudes in a cycle on the complex plane, for four different sets of (*M*_0_, *B*). The other parameters are taken to be the same as in Fig. 2.

To further confirm the above general discussion, we plot the trajectories of *r*_↑↑_ for four different representative cases in Fig. 3(a–d). The reflection amplitudes always move on the unit circle around the origin on the complex plane, as discussed above. In (a), *r*_↑↑_ goes around the origin counterclockwise once in a cycle. This makes *φ*_*s*_ jump 2*π*, as shown in Fig. 2(a). As a result, *W*_↑_ = 1, in agreement with the spin Chern number *C*_↑_ = 1. In (b) and (c), the trajectory of *r*_↑↑_ starts from one point, travels to another, and then returns. While this gives small jumps of the argument *φ*_↑_ of *r*_↑↑_ near *k*_*y*_ = 0, the overall increment of *φ*_↑_ from *k*_*y*_ = −∞ to *k*_*y*_ = +∞ is zero. The orbit of *r*_↑↑_ does not travel around the origin once. Therefore, the winding number is zero *W*_↑_ = 0, in agreement with *C*_↑_ = 0. In (d), *r*_↑↑_ goes around the origin once in the clockwise direction. Therefore, *W*_↑_ = −1, in agreement with *C*_↑_ = −1. Actually, these results are independent on any other parameters, such as *ωt* and *γ*_0_. The winding numbers are fully equal to the spin Chern numbers from the calculated trajectories of *r*_↑↑_.

In a real system, there is always a finite cutoff on the momentum *k*_*y*_. From Fig. 2, we can see that the argument of the complex reflection amplitude changes value only near *k*_*y*_ = 0. It quickly becomes flat with increasing or decreasing *k*_*y*_ away from *k*_*y*_ = 0. Therefore, the increment of the argument would not be affected by the cutoff on *k*_*y*_ as long as the size of the system is sufficiently large in the *y* direction. Here, we want to emphasize that only the adiabatic condition is essentially the cornerstone of the entire formulation. Other assumptions or approximations, such as the existence of *ac* electric filed in the electrode and barriers, the details of the barriers, and a finite cutoff on the momentum *k*_*y*_ and so on, have no effect on the main results. In addition, similarly to ref. 31, one can also include the Rashba spin-orbit coupling into *H*_QSH_, and find that it leads to small deviation of the spin Hall conductivity from the integer-quantized value, in the second order of *R*/*v*_F_ with *R* as the strength of the Rashba spin-orbit coupling.

### Experimental Measurement of The Ac QSH Effect

Now we propose a method to experimentally measure the *ac* spin Hall conductivity by electrical means. As shown in Fig. 1(b), we suggest to use ferromagnetic metals instead of the electrode leads, with their magnetic moment aligned along the *z* axis. The ferromagnets are assumed to have a length *L*, in the *x* direction, much greater than the spin diffusion length ${\ell }_{{\rm{D}}}$. As demonstrated above, the spin current generated is topological and insensitive to the material parameters of the electrode. When the *ac* electric field *E*(*t*) is applied, a pure *ac* spin current density *σ*_SH_ *E*(*t*) will flow out of the source ferromagnet and into the drain ferromagnet. The principle of the proposed method can be explained as follows. The amplitude of the spin current decays into the electrodes within about a spin diffusion length ${\ell }_{{\rm{D}}}$, and its spin-up and spin-down components recombine to cancel each other. Since the majority-spin and minority-spin bands of a ferromagnet have different 2D conductivities, *σ*_M_ and *σ*_m_, the voltage drops (strictly speaking, drops of the electrochemical potential) in the two spin channels caused by the spin current are not equal in magnitudes, giving rise to a net *ac* electric voltage drop. If the voltages at the outside edges of the ferromagnetic electrodes are made equal through grounding or short-circuiting, a voltage difference *V*_SH_(*t*) will appear between the inner edges of the electrodes, which can be measured by using an *ac* voltage meter.

The measured spin Hall voltage is dependent of impurity scattering and spin relaxation, which we now need to take into account. In the ferromagnetic electrodes, the spin diffusion length ${\ell }_{{\rm{D}}}$ is assumed to be much smaller than the length *L* of the electrodes. Therefore, the electronic transport in the electrodes is in the diffusive regime, rather than in the ballistic regime. In the diffusive regime, the scattering matrix formula is impracticable for analytical calculation. Instead, we employ the semiclassical spin diffusion equation for a ferromagnetic metal, which in the adiabatic regime can be written as ref. 32

9 $${\nabla }^{2}{\mu }_{s}(x,t)=\frac{{\mu }_{s}(x,t)-{\mu }_{-s}(x,t)}{{l}_{s}^{2}}.$$

Here, ${l}_{s}={v}_{{\rm{F}}}\sqrt{{\tau }_{s}{\tau }_{\uparrow \downarrow }/3}$, where *τ*_*s*_ and *τ*_↑↓_ are the electron non-spin-flip and spin-flip relaxation times, *μ*_*s*_(*x*, *t*) is the spin-dependent electrochemical potential for spin-up (*s* = ↑) and spin-down (*s* = ↓) electrons, respectively, and *v*_F_ is the Fermi velocity in the ferromagnets. Here, the adiabatic condition is $\omega {\tau }_{{\rm{D}}}\ll 1$, where *ω* is the frequency of the *ac* electric field, and ${\tau }_{{\rm{D}}}={\ell }_{{\rm{D}}}/{v}_{{\rm{F}}}$ is the spin relaxation time in the ferromagnetic metal. The usually small spin dependence in the Fermi velocity has been neglected^33, 34^. The spin-dependent electrical current is given by

10 $${{\mathscr{J}}}_{s}(x,t)=-{\sigma }_{s}\nabla {\mu }_{s}(x,t),$$

where ${\sigma }_{s}={e}^{2}{\tau }_{s}{k}_{{\rm{F}}}^{3}\mathrm{/6}{\pi }^{2}m$ is the Drude conductivity.

We consider first the drain ferromagnetic electrode, as the right electrode. In this case, the topological pure spin current *σ*_SH_ *E*(*t*) flows from the QSH material into the electrode. The boundary condition at the left edge (*x* = 0) of the electrode is given by

11 $${{\mathscr{J}}}_{\uparrow }(\mathrm{0,}\,t)=-{{\mathscr{J}}}_{\downarrow }(\mathrm{0,}t)=\frac{e}{\hslash }{\sigma }_{{\rm{SH}}}E(t),$$

while that at the right edge (*x* = *L*) reads

12 $${\mu }_{\uparrow }(L,t)={\mu }_{\downarrow }(L,t)=0,$$

since the right edge is grounded. From Eqs (9–12), one can readily obtain for the spin-dependent electrochemical potential

13 $${\mu }_{\uparrow }(x,t)=-{\mu }_{0}\frac{{\ell }_{{\rm{D}}}^{2}}{{l}_{\uparrow }^{2}}{e}^{-x/{\ell }_{{\rm{D}}}},$$

14 $${\mu }_{\downarrow }(x,t)={\mu }_{0}\frac{{\ell }_{{\rm{D}}}^{2}}{{l}_{\downarrow }^{2}}{e}^{-x/{\ell }_{{\rm{D}}}},$$

with

$${\mu }_{0}(t)=\frac{e}{\hslash }{\sigma }_{{\rm{SH}}}E(t){\ell }_{{\rm{D}}}\,(\frac{1}{{\sigma }_{\uparrow }}+\frac{1}{{\sigma }_{\downarrow }}),$$

where ${\ell }_{{\rm{D}}}^{-2}={l}_{\uparrow }^{-2}+{l}_{\downarrow }^{-2}$ and ${\ell }_{{\rm{D}}}$ is the spin-diffusion length. The electric voltage *μ*(*x*, *t*) in the ferromagnet is the average of spin up and down chemical potentials, $\mu (x,t)=\frac{1}{2}[{\mu }_{\uparrow }(x,t)+{\mu }_{\downarrow }(x,t)]$.

The electric voltage in the source electrode can be solved similarly. We find that the electric voltage at the right edge of source electrode is equal to that at the left edge of drain electrode in magnitude, but with an opposite sign. As a result, the electric voltage difference between the two inside edges of the two electrodes is *V*_SH_(*t*) = 2*μ*(0, *t*), which can be derived to be

15 $${V}_{{\rm{SH}}}(t)=\frac{e}{\hslash }{\sigma }_{{\rm{SH}}}E(t){\ell }_{{\rm{D}}}(\frac{1}{{\sigma }_{{\rm{m}}}}-\frac{1}{{\sigma }_{{\rm{M}}}}).$$

Here, *σ*_M_ and *σ*_m_ are the majority-spin and minority-spin conductivities, as mentioned above. Therefore, by measuring the electric voltage difference *V*_SH_(*t*), the spin Hall conductivity *σ*_SH_ can be determined.

Finally, it is worthwhile to discuss the experimental conditions to detect the *ac* QSH effect. Let us assume that a HgTe quantum well with thickness *d* = 7.0 nm is used as the QSH material, whose bulk band gap is ${{\rm{\Delta }}}_{{\rm{gap}}}\simeq 20\,{\rm{meV}}$ ^35^. The spin diffusion length in a ferromagnetic metal is typically ${\ell }_{{\rm{D}}}\simeq 0.1\,{\rm{um}}$ ^32^. The typical Fermi velocity of a metal is ${v}_{{\rm{F}}}\simeq {10}^{6}\,{\rm{m}}/{\rm{s}}$ ^36^. As a result, the spin relaxation time is estimated to be ${\tau }_{{\rm{D}}}={\ell }_{{\rm{D}}}/{v}_{{\rm{F}}}\simeq {10}^{-13}\,{\rm{s}}$. To ensure the system in the adiabatic transport regime, we need to require $\hslash \omega \ll {{\rm{\Delta }}}_{{\rm{gap}}}$ and $\omega {\tau }_{{\rm{D}}}\ll 1$, so that the frequency needs to satisfy $\omega \ll {10}^{13}\,{\rm{Hz}}$. In addition, the length of the ferromagnetic electrodes needs to satisfy $L\gg {\ell }_{{\rm{D}}}\simeq 0.1\,{\rm{um}}$. All these conditions should be easily accessible experimentally. If Mn films of 1 nm are chosen to be the ferromagnetic electrodes, the 2D majority-spin and minority-spin conductivities are estimated as *σ*_M_ = 10*σ*_m_ and ${\sigma }_{{\rm{m}}}\simeq 7\times {10}^{-4}/{\rm{\Omega }}$ ^36^. When the electric field is taken to be ${E}_{0}\simeq 1\,{\rm{mV}}/{\rm{nm}}$, the magnitude of the electric voltage difference is estimated to be ${V}_{{\rm{SH}}}\simeq {E}_{0}{\ell }_{{\rm{D}}}/20=5\,{\rm{mV}}$, which is readily measurable experimentally.

## Conclusion

In this work, we have developed an analytical theory of the *ac* QSH effect by using the time-dependent scattering matrix method. We proposed the setup illustrated in Fig. 1, where the *ac* electric field is applied to the bulk of the QSH material without coupling to the edge states, in order to demonstrate the fact that the *ac* QSH effect is a bulk transport phenomenon, essentially different from the *dc* QSH effect. In such a setup, the QSH effect occurs as a quantum pumping effect driven by the time-dependent *ac* electric field, and vanishes for an exactly static electric field with *ω* = 0. The resulting *ac* spin current flowing from the QSH material into an electrode is linked to the winding numbers of the reflection matrix of the electrode, which also equal to the spin Chern numbers of the QSH system. For low frequencies, the present scattering matrix theory is in agreement to the calculation based upon the Kubo linear-response theory. The two theories have their respective advantages, and are mutually complimentary. A possible way to observe the *ac* QSH effect experimentally was also suggested.
